# Editorial: Adverse Reactions to Biomaterials: State of the Art in Biomaterial Risk Assessment, Immunomodulation and *in vitro* Models for Biomaterial Testing

**DOI:** 10.3389/fbioe.2019.00015

**Published:** 2019-02-11

**Authors:** Nihal Engin Vrana, Amir M. Ghaemmaghami, Pinar Zorlutuna

**Affiliations:** ^1^Protip Medical, Strasbourg, France; ^2^Faculté de Chirurgie Dentaire, Université de Strasbourg, Fédération de Médecine Translationnelle de Strasbourg, Fédération de Recherche Matériaux et Nanosciences Grand Est, Strasbourg, France; ^3^Immunology and Immuno-Bioengineering Group, Faculty of Medicine and Health Sciences, School of Life Science, University of Nottingham, Nottingham, United Kingdom; ^4^Bioengineering Graduate Program, University of Notre Dame, Notre Dame, IN, United States; ^5^Department of Aerospace and Mechanical Engineering, University of Notre Dame, Notre Dame, IN, United States

**Keywords:** biomaterials, immunomodulation, *in vitro* models, risk assessment, adverse reactions

The recent advances in polymer chemistry (an ever increasing number of new polymers or derivatives of existing polymers with new properties) (Liu et al., 2018), materials science (metamaterials specifically designed for a given application, hybrid materials and highly controlled composite structures at nano/micro levels, materiomics) (Kowalski et al., 2018), biotechnology (new gene editing technologies for facilitating natural biomaterial production, accelerated rate of discovery, and isolation of functional natural biomaterials) (Sakar and Baker, 2018) and manufacturing techniques (3D printing, bioprinting, controlled self-assembly methods) (Nagarajan et al., 2018) have provided a significant boost in our capabilities to offer new solutions for debilitating chronic diseases in the form of implantable devices and regenerative medicine products. However, this rapid expansion in the biomaterials toolkit unfortunately comes at a cost; a considerable increased risk of adverse reactions to implanted biomaterials which includes allergies, chronic inflammation, susceptibility to infection, collateral tissue damage, and loss of functionality due to immune reactions. Moreover, the highly personalized nature of immune reactions needs to be taken into account while assessing the use of new biomaterials. These concerns have created a general reticence in the medical device industry for the utilization of novel biomaterials and complex, multi-material structures which significantly hinders the advances in the field and also decelerates the introduction of new and potentially transformative technologies to the healthcare system.

The potential ways out of this conundrum are (i) to improve our capacities in the risk assessment of biomaterials by developing novel *in vitro* testing systems which can provide more relevant and in-depth information about cell/tissue/organ-biomaterial interactions, preferably in a personalized manner (i.e., using patients' own cells); (ii) to develop new technologies to control the interface between the implanted materials and the host tissues where immune reactions can be either attenuated or directed toward expected outcomes (immunomodulation and integration) (iii) to achieve systems level understanding of personalized response to biomaterials using the recent advances in high throughput analysis methods available (immunoprofiling).

Being actively involved in the development of such technologies (Ellis et al., 2017; Rostam et al., 2017; Kubon et al., 2018), our motivation for this research topic was to bring together a group of high quality articles that provide insights on responses to biomaterials, immunomodulation and *in vitro* model systems. The review articles in the topic cover: skin substitutes with immunomodulatory components and skin models with their use in biomaterial assessment (Savoji et al.), haemocompatibility of biomaterials and the analyses of interactions of biomaterials with human blood (Weber et al.), liver- and lung-on-a-chip systems for biomaterial testing and related *in silico* models (Nikolic et al.), and an overview of production and development of a medical grade biomaterial and its derivatives using Hyaluronic acid as an example (Huerta-Ángeles et al). The original research articles in the research topic covered different aspects of immune reaction and immunomodulation such as: the effect of photocrosslinked Gelatin hydrogels on Tumor Necrosis Factor Alpha (TNF-α) secretion by primary human mononuclear cells and underlying mechanisms (Donaldson et al.), the incorporation of macrophages for immune assisted tissue engineering within hydrogel structures in co-culture and tri-culture configurations (Barthes et al.), the multinucleated giant cell induction by collagen based membranes *in vivo* and its potential ramifications (Al-Maawi et al). The upcoming, materiomics-based biomaterial selection methodologies were represented by the detection of distinct biomaterial topographies that can be used for Fibroblastic Reticular Cell differentiation by TopoChip system (Vasilevich et al.), whereas utilization of an advanced biomechanical testing system that can mimic natural loading conditions in the articular cartilage was used to demonstrate the potential ways to optimize polymeric composite scaffold mechanical properties for better integration (Gasik et al). Finally, as an example of a potential parameter to compare biomaterial properties and evaluate coatings for decision-making purposes, a methodology based on zeta potential measurements was presented (Ferraris et al.).

The key to better harness the innovations in biomaterial and biomedical device fields is to establish the necessary methodologies and model systems for their risk assessment, validation, and testing. Recent success and ongoing efforts in developing technologies for immune engineering, personalized biomaterials and personalized *in vitro* testing platforms will bring forth the solutions that can improve the quality of life and life expectancy even further in twenty-first century.

## Author Contributions

NV, AG, and PZ equally contributed to the preparation of the manuscript. The submitted version is revised and confirmed by all authors.

### Conflict of Interest Statement

The authors declare that the research was conducted in the absence of any commercial or financial relationships that could be construed as a potential conflict of interest.
